# The Effects of ROCK Inhibitor on Prevention of Dexamethasone-Induced Glaucoma Phenotype in Human Trabecular Meshwork Cells

**DOI:** 10.1167/tvst.12.12.4

**Published:** 2023-12-05

**Authors:** Tilahun Ayane Debele, Zachary F. Mount, Yong Yuan, Winston W.-Y. Kao, Yoonjee C. Park

**Affiliations:** 1 Department of Chemical and Environmental Engineering, College of Engineering and Applied Science, University of Cincinnati, Cincinnati, OH, USA; 2 Department of Ophthalmology, College of Medicine, University of Cincinnati, OH, USA

**Keywords:** glaucoma, dexamethasone, ripasudil, co-delivery, F-actin, contractility

## Abstract

**Purpose:**

This study investigated the effects of dexamethasone (Dex) on human trabecular meshwork (TM) cells, a model of glucocorticoid-induced glaucoma, and evaluated the impact of ripasudil (Rip) as a co-delivery or sequential dosing strategy.

**Methods:**

In vitro experiments were conducted to assess the effects of Dex and Rip on TM cells. Confocal microscopy was used to evaluate the impact of Dex and Rip on F-actin staining signals. Contractility of the TM cells upon Dex and Rip treatment mimicking co-delivery and sequential delivery was quantified using collagen gel contraction assay. Transepithelial electrical resistance (TEER) values and fluorescein isothiocyanate (FITC)–dextran permeability were also measured to assess the impact of Dex and Rip on TM cells.

**Results:**

Dex and Rip did not exhibit cytotoxicity at the maximum tested concentration (20 µM). Dex-treated TM cells exhibited higher F-actin staining signals compared to controls, which were reduced when co-treated with Rip. Rip inhibited Dex-induced collagen gel contraction activity in both co-delivery and sequential treatments. Dex resulted in increased TEER values as the dose increased, whereas TEER values were maintained when co-treated with Rip.

**Conclusions:**

Co-delivery of Rip has the potential to prevent glaucoma symptoms when patients are treated with Dex. This study highlights the importance of identifying strategies to reduce the side effects of prolonged use of glucocorticoids, such as Dex, in the treatment of various diseases.

**Translational Relevance:**

This study demonstrates the potential of co-delivering ripasudil with dexamethasone to mitigate glucocorticoid-induced ocular hypertension and a secondary glaucoma that resembles primary open-angle glaucoma, providing insights for the development of novel preventive strategies in clinical care.

## Introduction

Glaucoma is a condition in which the optic nerve of the eye becomes damaged, posing a significant risk for vision loss and eventual irreversible blindness if left untreated. Although several studies have shown that the largest risk factors of glaucoma are genetics, age, and elevated intraocular pressure (IOP), detailed mechanisms underlying glaucoma are not well known.^1^ One of the major risk factors is glucocorticoid (corticosteroid)-induced ocular hypertension.^2^

Dexamethasone sodium phosphate (Dex) is a synthetic glucocorticoid that is crucial in the treatment of eye diseases due to its wide-ranging anti-inflammatory effects.^3^^,^^4^ Current standard of care for chronic posterior eye diseases is intravitreal dexamethasone implant (Ozurdex) injections. However, the IOP in ∼25% of patients who received Ozurdex significantly increased and peaked at ∼8 weeks. The elevated IOP is primarily caused by increased outflow resistance of aqueous humor (AH), which can ultimately result in optic nerve damage and blindness.^5^^,^^6^ Several studies have shown that Dex increases the AH outflow resistance via ultrastructural and biochemical changes in trabecular meshwork (TM) cells and/or Schlemm's canal endothelial (SCE) cells.^7^^–^^10^ For example, Clark et al.^7^ observed actin microfilament reorganization, referred to as cross-linked actin networks (CLANs), in the TM cells when exposed to glucocorticoid. Yuan et al. also reported Dex-induced stress fiber rearrangement in TM cells forming CLANs through the ROR2/RhoA/ROCK signaling axis.^5^ Rearrangement of the actin cytoskeleton can affect contractility through its association with myosin light-chain phosphorylation. Fujimoto et al.^44^ also showed Dex increases RhoA activity and reduces outflow facility in both TM and SCE cells. Other studies revealed that altered gene and protein expression, excessive extracellular matrix (ECM) production, and reduced TM phagocytic function seen in the Dex-treated TM cells can be participating in the pathogenesis of Dex-induced glaucoma.^11^^–^^16^

In 2014, ripasudil hydrochloride hydrate (Rip), a Rho-associated coiled-coil containing protein kinase (ROCK) inhibitor was approved for the treatment of glaucoma.^17^ Rip is a small-molecule ROCK inhibitor developed by Kowa Company (Nagoya, Japan). Rip decreases IOP through the direct action of relaxation on the TM cells causing increased permeability, which in turn decreases the resistance of AH outflow. Hence, a possible mechanism for reducing IOP is by inhibiting ROCK downstream effector proteins that regulate TM cell morphology such as actin cytoskeleton organization.^18^^–^^20^

We hypothesized that that Rip co-delivery with Dex or sequential addition of Rip to Dex-treated TM cells can prevent or reverse Dex-induced phenotypic changes in TM cells. Several studies have demonstrated that cultured human TM cells share many properties with human TM cells in vivo, and they are commonly used as a model to study the biological effects of glaucoma.^21^^–^^23^ Thus, to test our hypothesis, we examined the effects of Rip co-delivery with Dex or as a sequential treatment on contractility, permeability, and stress fiber levels of TM cells. To the best of our knowledge, this study is the first to demonstrate the effects on TM cells of Dex with Rip as a co-delivery or sequential addition. In this study, we also investigated the effects of Dex doses on TM cell viability, filamentous actin (F-actin) cytoskeleton reorganization, collagen gel matrix contraction, and cell membrane integrity, in the presence or absence of Rip. The investigation tested our hypothesis that co-delivery or sequential delivery of Rip with Dex maintains the normal physical phenotypes of TM cells. Furthermore, this study suggests that these dosing strategies have the potential to maintain AH outflow akin to normal conditions and to mitigate high IOP formation. Overall, this study provides new insights into the potential use of Rip in the prevention of glaucoma.

## Methods and Materials

### Materials

Dex was purchased from The Lab Depot (Dawsonville, GA), and ripasudil was purchased from AdooQ Bioscience (Irvine, CA). Phosphate-buffered saline (10×), Alexa Fluor 555, phalloidin, and MTT (3-[4,5-dimethylthiazol-2-yl]-2,5-diphenyltetrazolium bromide) reagents were purchased from Thermo Fisher Scientific (Waltham, MA). Cell contraction assays were purchased from Cell Biolabs (San Diego, CA). Fluorescein isothiocyanate (FITC)–dextran was purchased from Sigma-Aldrich (St. Louis, MO). Human trabecular meshwork (HTM) cell growth medium and HTM cells, which had been isolated from normal healthy human adult eyes, were purchased from Cell Applications (San Diego, CA). Dulbecco's modified Eagle's medium (DMEM)/Ham's F-12 50/50 mix was purchased from Corning Life Science (Tewksbury, MA). Fetal Bovine Serum–Premium Select was purchased from Bio-Techne (Minneapolis, MN).

### Human TM Cell Culture

Per the manufacturer's instructions, the TM cells were cultured in human TM cell growth medium, which was ready to use, and they were incubated at 37°C with 5% CO_2_,^24^ consistent with consensus recommendations for such culture.^24^ TM cells from passages three to eight were used in this study.

### In Vitro Cytotoxicity Assay

To evaluate the cytotoxicity of Dex, Rip, and Dex+Rip on TM cells in vitro, an MTT assay was performed. TM cells were seeded in 96-well plates at a density of 5 × 10^3^ cells per well and incubated for 24 hours to promote cell attachment. The cells were then exposed to a range of concentrations of Dex (10 nM, 100 nM, 1 µM, 10 µM, and 20 µM), Rip (0.5 µM, 1 µM, 5 µM, 10 µM, and 20 µM), and Dex+Rip (10-nM Dex + 10-µM Rip, 100-nM Dex + 10-µM Rip, 1-µM Dex + 10-µM Rip, 10-µM Dex + 10-µM Rip, and 20-µM Dex + 10-µM Rip), and they were incubated at 37°C with 5% CO_2_ for 6 days. Every 2 days, the medium was changed to their respective doses. After 6 days of incubation, 20 µL of MTT (5 mg/mL) solution was added, and the cells were further incubated for 4 hours. The media in the wells were then removed, and 100 µL of dimethyl sulfoxide (DMSO) was added to dissolve the internalized purple formazan crystals. The absorbance was measured at a test wavelength of 570 nm using an enzyme-linked immunosorbent assay (ELISA) reader (Power Wave XS; BioTek, Winooski, VT). The means and standard deviations (SDs) of cell viability (%) were calculated for the measurements of four replicates (*n* = 4). Finally, the relative cell viability (%) was calculated using the following equation:

$$\begin{array}{ccc} & & \;\mathrm{Cell}\;\mathrm{viability}\;\left(\%\right)\\ & & \;=\;\frac{\mathrm{Absorbance}\;\mathrm{of}\;\mathrm{test}\;\mathrm{cells}}{\mathrm{Absorbance}\;\mathrm{of}\;\mathrm{controlled}\;\mathrm{cells}}\times \;100 \end{array}$$

### Lactate Dehydrogenase Cytotoxicity Assay

The effects of Dex, Rip, and Dex+Rip on TM cell toxicity were investigated using the CyQUANT LDH Cytotoxicity Assay (Invitrogen C20300; Thermo Fisher Scientific) according to the manufacturer's recommendations. Briefly, TM cells were plated in 96-well plates at a density of 1 × 10^4^ cells/well and cultured overnight to ensure attachment to the plates. The cells were then exposed to a range of concentrations of Dex (10 nM, 100 nM, 1 µM, 10 µM, and 20 µM), Rip (0.5 µM , 1 µM, 5 µM, 10 µM, and 20 µM), and Dex+Rip (10-nM Dex + 10-µM Rip, 100-nM Dex + 10-µM Rip, 1-µM Dex + 10-µM Rip, 10-µM Dex + 10-µM Rip, and 20-µM Dex + 10-µM Rip) and incubated at 37°C with 5% CO_2_ for a total of 6 days. After 6 days of treatment, the cell culture medium was collected and used for the LDH assay according to the manufacturer’s recommendation. Briefly, 50 µL of each sample medium (drug-treated, spontaneous LDH activity controls, and maximum LDH activity controls) was transferred to 96-well flat bottom plates in quartet wells. Then, 50 µL of reaction mixture was added to each sample well and mixed by gentle tapping. The plate was incubated at room temperature for 30 minutes under light protection. After 30 minutes of incubation, 50 µL of stop solution was added to each sample well and mixed by gentle tapping. The absorbance was then measured at a test wavelength of 490 nm and reference wavelength of 680 nm. To determine LDH activity, the absorbance value at 680 nm (background) was subtracted from the 490-nm absorbance before calculation of the percent cytotoxicity. TM cells were plated in quartet well for spontaneous LDH activity controls (10 µL of sterile, ultrapure water was added to each set of wells) and maximum LDH activity controls (10 µL of 10× lysis buffer was added to each well).

The percent cytotoxicity was calculated by using the following formula:

$$\begin{array}{ccc} & & \;\%\;\mathrm{Cytotoxicity}\;\\ & & \;=\frac{\;\mathrm{Drug}\;\mathrm{treated}\;\mathrm{LDH}\;\mathrm{activity}\;-\;\mathrm{spontaneous}\;\mathrm{LDH}\;\mathrm{activity}\;}{\mathrm{Maximum}\;\mathrm{LDH}\;\mathrm{activity}-\;\mathrm{spontaneous}\;\mathrm{LDH}\;\mathrm{activity}}\times \;100 \end{array}$$

The means and SDs were calculated based on the results obtained from quartet wells for each condition (*n* = 4).

### F-Actin Cytoskeleton

To investigate the effect of Dex, Rip, and Dex+Rip on the F-actin cytoskeleton of TM cells, a modified version of a previously published method was used.^25^ Briefly, TM cells were seeded in 12-well chamber slides at a density of 1.5 × 10^4^ cells per well in DMEM containing 10% fetal bovine serum. After overnight culture, various concentrations of Dex (10 nM, 100 nM, 10 µM), Rip (10 µM), and Dex+Rip (10-nM Dex + 10-µM Rip, 100-nM Dex + 10-µM Rip, and 10-µM Dex + 10-µM Rip) were added to the culture wells. DMEM medium was used as a control vehicle, and the media with respective drug doses were changed every 2 days. After 6 days of treatment, the drug solutions were removed, and the cells were washed with phosphate-buffered saline (PBS). The cells were then fixed with 4% paraformaldehyde in PBS for 15 minutes and washed with PBS. Subsequently, the cells were permeabilized with 0.2% Triton X-100 in PBS for 15 minutes at room temperature and washed again with PBS. The F-actin was labeled with 0.05 mg/mL Alexa Fluor 555 phalloidin for 1 hour at room temperature. After they were washed with PBS, the cells were incubated with 4′,6-diamidino-2-phenylindole (DAPI) for 10 minutes. The cells were then mounted with a commercial mounting medium (Invitrogen SlowFade Gold Antifade Mountant; Thermo Fisher Scientific), and the images were observed using a Leica STELLARIS 8 Confocal Microscope (Leica, Wetzlar, Germany). ImageJ (National Institutes of Health, Bethesda, MD) was used to analyze each image. Briefly, all selected representative images (five images for each condition) were imported into ImageJ. Then, F-actin staining signals were selected (polygon selection) for each image and changed to 8 bits (image → type → 8-bit). Finally, the mean gray value of each image was measured (analyze → set measurement → measure), and the averages and SDs were calculated based on the five representative images for each condition (*n* = 5).

### Collagen Gel Matrix Contraction

The working solution of collagen gel was prepared in accordance with the manufacturer's instructions. To achieve this, 9.54 mL of collagen solution and 2.46 mL of 5× DMEM medium were mixed in a cold sterile tube, following the manufacturer's instructions. Next, 340 µL of neutralization solution was added to the mixture, which was immediately stirred. The resulting collagen gel working solution was kept on ice until required. In this experiment, a two-step collagen contraction model was employed. Initially, TM cells were harvested and resuspended in the desired medium at a concentration of 2 to 5 × 10^6^ cells/mL. Following this, the collagen lattice was formed by combining two parts of cell suspension with eight parts of cold collagen gel working solution. The resulting cell–collagen mixture (0.5 mL) was added to each well in a 24-well plate and incubated for 1 hour at 37°C in 5% CO_2_ for collagen polymerization. Subsequently, 1.0 mL of culture medium or dosed drug was added on the top of each collagen gel lattice. The TM cell–collagen gel matrix was treated with serial concentrations of Dex (10 nM, 100 nM, and 10 µM) in the presence or absence of Rip (10 µM) for 72 hours, followed by 24 hours post-release. Each condition was triplicated (*n* = 3). The collagen gel was photographed, and ImageJ was used to measure the collagen gel area. Briefly, all of the selected representative collagen gel images (two images for each condition) were imported into ImageJ. Then, collagen gel was selected (polygon selection) and the area of each image was measured (analyze → set measurement → measure). Based on the actual area of the 24-well plate (190 mm^2^), the collagen gel area was calculated.

### Transepithelial Electrical Resistance Measurement of TM Cell–Collagen Gel Matrix

The transepithelial electrical resistance (TEER) values of the TM cell–collagen gel matrix were determined using a modified version of previously described methods.^25^^,^^26^ Briefly, a mixture of two parts of TM cell suspension (1 × 10^6^ cells/mL) and eight parts of cold collagen gel working solution was prepared. Then, 0.25 mL of the resulting TM cell–collagen mixture was added to a transwell polyester membrane insert (0.4-µm pore size and 6.5-mm diameter; Corning, Corning, NY) placed on a 24-well culture plate (5 × 10^4^ cells/insert) and incubated for 1 hour at 37°C with 5% CO_2_ for collagen polymerization. Following this, 0.25 mL of culture medium (DMEM) was added on top of each collagen gel lattice, and 1.2 mL of medium was added to the basal side (outside the membrane insert). To evaluate the effect of Dex, Rip, and Dex+Rip on TM barrier function, the TM cell–collagen matrix was treated with 0.25 mL of serial concentrations of Dex (10 nM, 100 nM, and 10 µM), Rip (10 µM), and Dex+Rip (10-nM Dex + 10-µM Rip, 100-nM Dex + 10-µM Rip, and 10-µM Dex + 10-µM Rip). Note that the media were changed every 2 days with the respective doses of Dex, Rip, or Dex+Rip. After 6 days of treatment, the TEER was measured using an EVOM volt/ohm meter (World Precision Instruments, Sarasota, FL) following the manufacturer's instructions. The TEER (*R*_cell_) values of the cell–collagen gel matrix were calculated after subtracting the blank resistance of the collagen gel without cells (*R*_blank_) from a measured TEER value. The percentage change of TEER values was calculated based on the TEER values of the control group (cell–collagen gel matrix cultured with medium only). The means and SDs were calculated based on three TEER measurements (*n* = 3).

$$\begin{array}{ccc} & & \;\mathrm{Percentage}\;\mathrm{change}\;\mathrm{of}\;\mathrm{TEER}\;\mathrm{value}\\ & & \;=\frac{\;R_{\mathrm{cell}}\;\mathrm{of}\;\mathrm{treated}\;\mathrm{group}}{R_{\mathrm{cell}}\;\mathrm{of}\;\mathrm{control}\;\mathrm{group}}\times \;100 \end{array}$$

### Permeability of TM Cell Monolayer

A 24-Transwell culture plate and polyethylene terephthalate (PET) membrane inserts with a pore size of 0.4 µm and a diameter of 6.5 mm were hydrated with enough medium to cover the insert for 15 minutes at room temperature. TM cells were then seeded onto the PET membrane insert at a density of 15,000 cells per well and incubated overnight at 37°C with 5% CO_2_. To assess the impact of Dex, Rip, and Dex+Rip on TM barrier function, serial concentrations of Dex (10 nM, 100 nM, and 10 µM), Rip (10 µM), and Dex+Rip (10-nM Dex + 10-µM Rip, 100-nM Dex + 10-µM Rip, and 10-µM Dex + 10-µM Rip) were added in volumes of 250 µL and 500 µL to the apical compartment of the insert and the basal compartment of the 24-well plates, respectively. The media in both compartments were replaced every 2 days throughout the duration of the assay. On day 6, the basal and apical compartments were washed with PBS, and 500 µL of 50-µM FITC–dextran in DMEM was added to the basal compartment, and 250 µL of DMEM was added to the apical compartment. The cultures were then incubated for 1 hour at 37°C to allow the FITC–dextran to diffuse across the cellular monolayer and the membrane. After 1 hour, the inserts were removed from the solution to halt the diffusion of FITC–dextran. A sample of 100 µL was collected from the apical compartment of the insert, transferred to an Eppendorf tube, and mixed thoroughly. The solution for each condition was then transferred to a black, round-bottom, 96-well ultraviolet (UV) plate for analysis using a UV–visible spectrophotometer (SpectraMax; Molecular Devices, San Jose, CA). The relative fluorescence intensity of FITC–dextran was measured using a multimode plate reader with an excitation wavelength of 490 nm and an emission wavelength of 530 nm. The means and SDs for FITC–dextran permeability were calculated based on the four experiments and two replicate fluorescence intensity measurements (*n* = 4).

### Statistical Analyses

Each experiment was conducted in triplicate or greater, and the statistical significance of the results was determined using a one-tailed Student's *t*-test. Results with *P* < 0.05 were considered statistically significant. The significance level is represented using asterisks as follows: *P < 0.05, **P < 0.01, and ***P < 0.001. The calculated *P* values for all experiments are included in the supplementary data (Supplementary Tables S1–S5).

## Results

### In Vitro Cytotoxicity of Dex, Rip, and Dex+Rip on TM Cells

**Figure 1.**
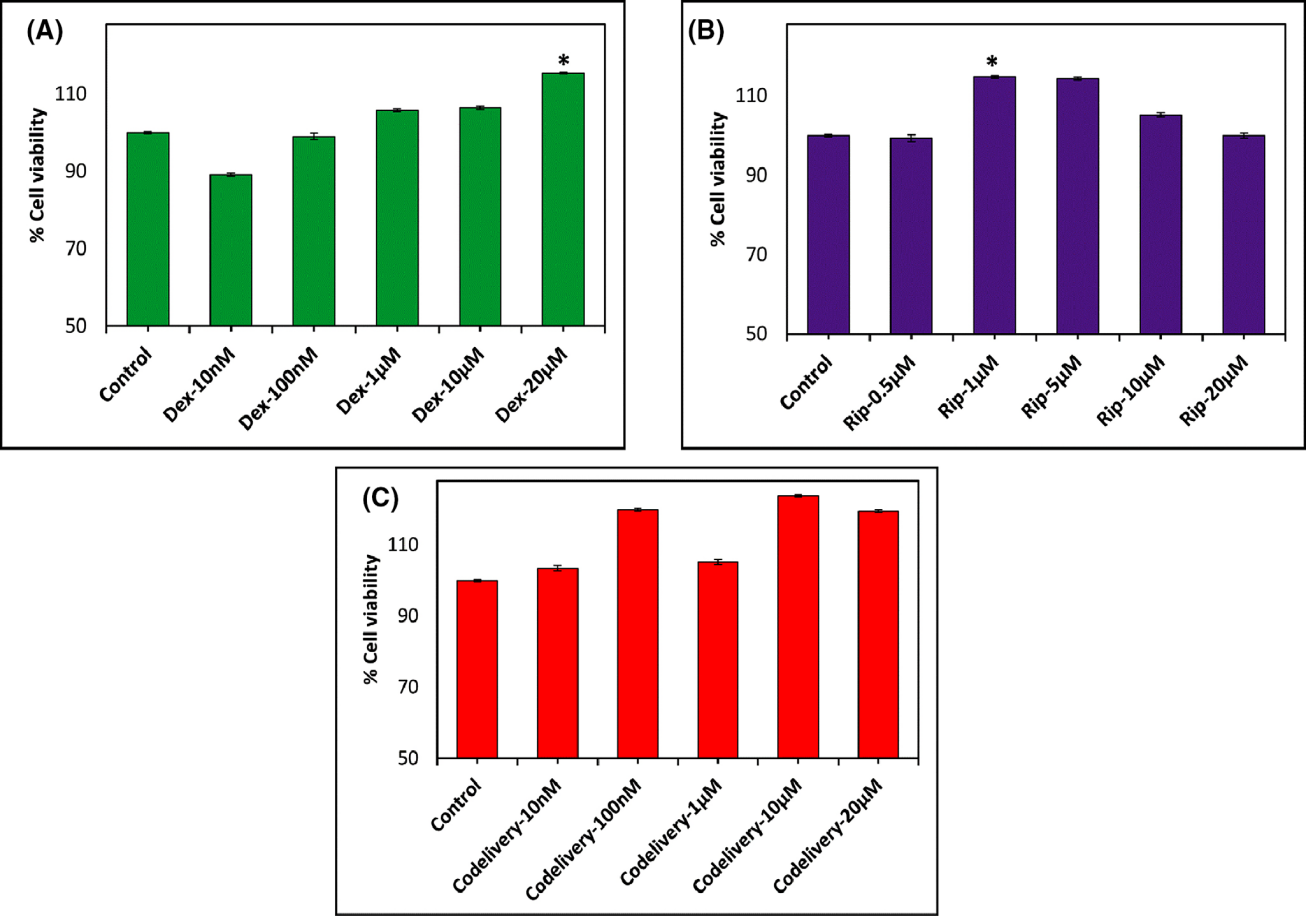
Dose-dependent cytotoxicity results of Dex (**A**), Rip (**B**), and co-delivery (Dex+Rip) (**C**) on TM cells as determined by MTT assays (*n* = 4). In the co-delivery experiments, an equal concentration of Rip (10-µM) was added to each serial concentration of Dex.

To assess the potential cytotoxic effects of Dex, Rip, and Dex+Rip, TM cells were exposed to a range of concentrations for 6 days. The MTT assay results, shown in Figure 1, indicate that more than 90% of TM cells remained viable following exposure to serial concentrations of Dex or Rip alone. We chose 10-µM Rip for the co-delivery of Dex and Rip because it showed cytotoxicity comparable to that of the control (∼100%), and 20-µM Rip started to show cell viability less than 100%, although it was not statistically significant. Several studies have shown that 10-µM Rip is effective in reversing the TM cell phenotypes caused by Dex.^27^^,^^28^ Additionally, co-treatment with 10-µM Rip did not affect the viability of TM cells at any of the tested concentrations of Dex, indicating viability that was the same or greater than 100%. Also previous studies have reported that the concentration is effective in reversing the TM cell phenotypes caused by Dex.^27^^,^^28^ Taken together, these results suggest that Dex, Rip, and Dex+Rip do not have any cytotoxic effects on TM cells at the tested concentrations.

**Figure 2.**
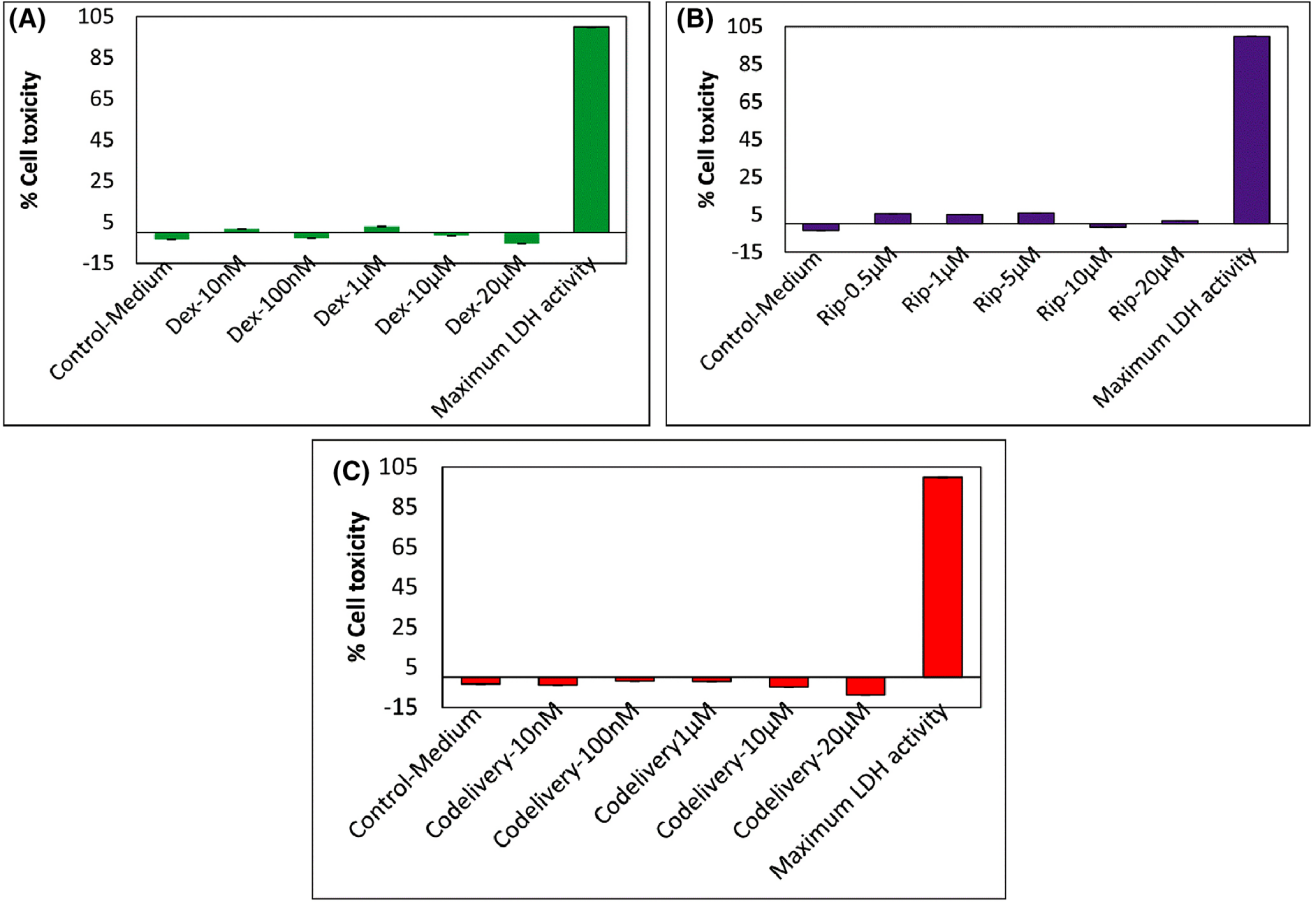
Dose-dependent cytotoxicity results of Dex (**A**), Rip (**B**), and co-delivery (Dex+Rip) (**C**) on TM cells as determined by the CyQUANT LDH Cytotoxicity Assay Kit (*n* = 4). An equal concentration of Rip (10-µM) was added to each serial concentration of Dex for the co-delivery experiments.

To support the MTT cytotoxicity results, we have also investigated the cytotoxicity effects of Dex, Rip, and Dex+Rip against TM cells using LDH cytotoxicity assay kits. LDH cytotoxicity (LDH release assay or LDH leakage assay) is the most widely used marker in cytotoxicity studies and is used to assess cell membrane integrity and cellular damage. This assay measures the release of LDH, a stable enzyme present in all cell types, into the surrounding culture medium or extracellular fluid as a result of cellular damage or cell death. Therefore, acute cytotoxicity can be measured by measuring the activity of these enzymes released into the culture supernatant. LDH oxidizes lactate to generate NADH, which then reacts with a certain dye to generate a yellow color. The intensity of the generated color correlates directly with the number of cells lysed, which is indicative of cytotoxicity. As shown in Figure 2, at all tested concentrations of Dex, Rip, and Dex+Rip, the percentage of cytotoxicity was negative to ∼5%, indicating less cellular damage due to the drugs.

### Effects of Dex, Rip, and Dex+Rip on TM Cell F-Actin Cytoskeleton

**Figure 3.**
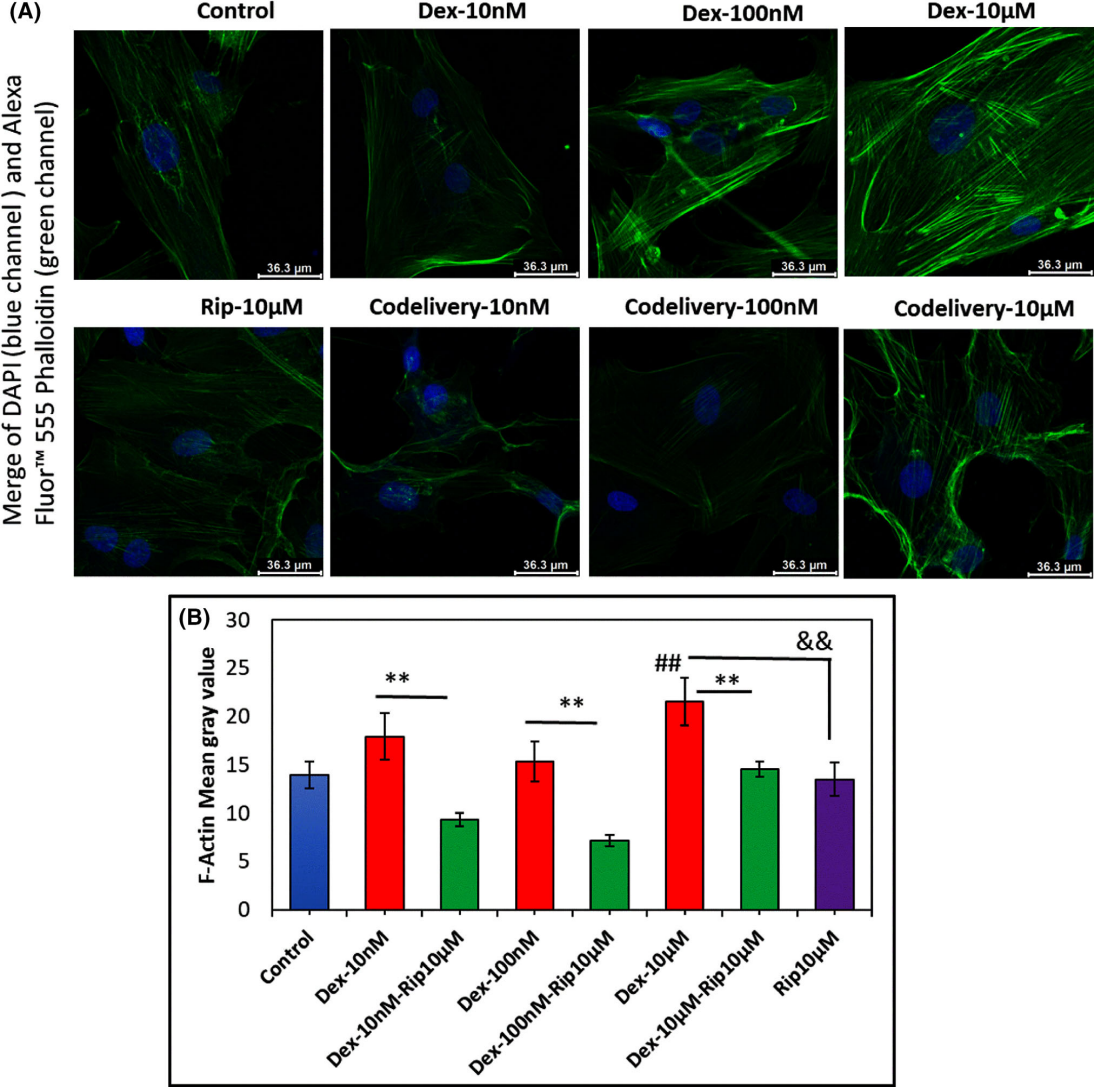
Representative image of TM cell cytoplasmic F-actin staining using Alexa Fluor 555 phalloidin. (**A**, **B**) Changes in F-actin distribution with a serial concentrations of Dex, Rip, and co-delivery of Dex &Rip. Gray values are means of cytoplasmic F-actin of Dex, Rip, and co-delivery of Dex+Rip dosed cultured TM cells for 6 days (*n* = 5). Distribution of F-actin (*green*) and DAPI (*blue*) in TM cells. In the co-delivery experiments, equal concentrations of Rip (10-µM) were added to each serial concentration of Dex. *Scale bar*: 36.3 µm. ^##^Control versus 10-µM Dex; ^&&^10-µM Rip versus 10-µM Dex.

This study evaluated the impact of Dex, Rip, and Dex+Rip on the organization of cytoplasmic F-actin, the primary component of the cytoskeleton, by staining with Alexa Fluor 555 phalloidin. Phalloidin selectively binds to the F-actin polymer in mammalian cells, allowing visualization of F-actin organization in TM cells. Confocal images in Figure 3A show that F-actin staining signals in Dex-treated TM cells were higher than those in the control group, especially for high doses of Dex. Furthermore, the polymerized F-actin stress fibers look straighter and more stretched out compared to those of the Rip-treated groups. Figure 3B shows that the F-actin mean gray value of Dex-treated TM cells was higher than that of the control group. Mainly, the mean gray value of TM cells treated with 10-µM Dex was significantly increased (^##^*P* = 0.008562) compared to the control group. In contrast, the F-actin mean gray value of TM cells treated with Dex+Rip was significantly lower than that of Dex-treated TM cells (**P < 0.01), suggesting that Dex and Rip have opposing effects on cytoskeleton rearrangement in TM cells. Specifically, Dex increases F-actin polymerization/stress fiber formation, whereas Rip initiates F-actin depolymerization.^29^ Interestingly, Rip alone did not reduce the signal intensity compared to the control (control vs. 10-µM Rip-10, *P* = 0.418). When both Dex and Rip were present, Rip further reduced the F-actin intensity (10-nM Dex + 10-µM Rip vs. 10-µM Rip, *P* = 0.0200; 100-nM Dex + 10-µM Rip vs. 10-µM Rip, *P* = 0.0025). However, for 10-µM Dex, the reduction was not significant (10-µM Dex-10 + 10-µM Rip vs. 10-µM Rip (*P* = 0.2834), suggesting that the stress fiber amounts at high doses of Dex could be too great to reverse.

### Collagen Gel Matrix Contraction

**Figure 4.**
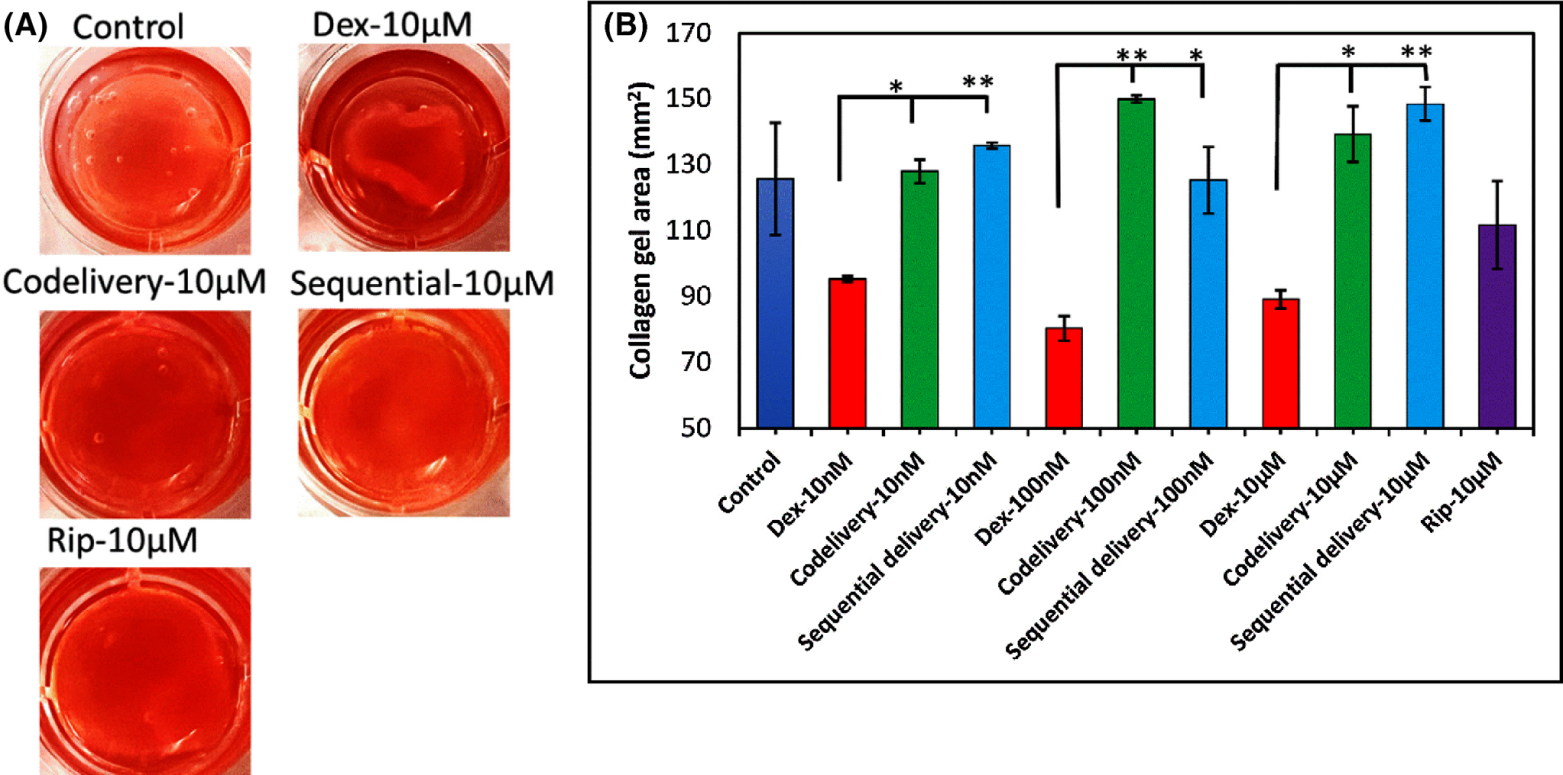
(**A**) Representative image of TM cell–collagen gel matrix treated with medium (control), Rip, Dex, or co-delivery (Dex+Rip) and sequential treatment (the concentration for co-delivery and sequential treatment is 10 µM). (**B**) Collagen gel area versus dosing conditions. ImageJ was used to analyze the collagen gel area (*n* = 3).

The present study investigated the effect of Dex, Rip, and Dex+Rip on collagen contraction mediated by TM cells. As shown in Figure 4, the addition of Dex to collagen gels containing TM cells induced a significant contraction compared to the control group. However, co-delivery (Dex+Rip) or sequential addition of Rip on a Dex-pretreated TM cell–collagen gel matrix significantly inhibited or reversed the collagen gel contraction activity of TM cells (10-nM Dex vs. 10-nM co-delivery, *P* = 0.012295; 100-nM Dex vs. 100-nM co-delivery, *P* = 0.003138; 10-µM Dex vs. 10-µM co-delivery, *P* = 0.028513; 10-nM Dex vs. 10-nM sequential delivery, *P* = 0.00105; 100-nM Dex vs. 100-nM sequential delivery, *P* = 0.0493; 10-µM Dex vs. 10-µM sequential delivery, *P* = 0.009163). Both co-delivery groups and sequential delivery groups at all three different doses showed no significant differences from the control, suggesting that Rip reversed the contraction to the control level. Although not statistically significant, there was a slight decrease in the collagen gel area of the TM cell–collagen gel matrix treated only with 10-µM Rip compared to control (control vs. 10-µM Rip, *P* = 0.3458). Also, no significant difference was observed between the Rip-only and the co-delivery or sequential delivery groups (all *P* > 0.05). The results indicate that Rip plays a significant role in reversing the contraction created by Dex in all conditions. In the absence of TM cells, 10-µM Dex (no cells) and 10-µM Rip (no cells) had no effect on gel contraction, as shown in Supplementary Figure S1, indicating that the contraction is due to the TM cells.

### TEER and Permeability

**Figure 5.**
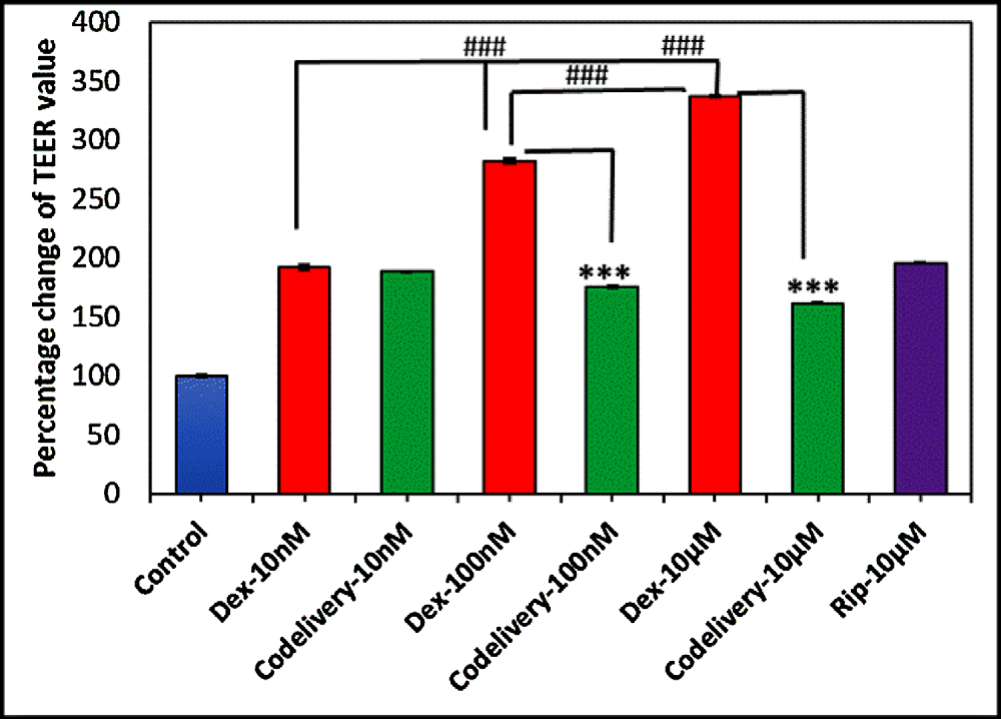
Percentage change of TEER values compared to control (*n* = 3). ^***(###)^*P* < 0.001 compared to the control groups or among the treatment groups.

For TEER measurement, TM cells grown in the collagen gel were tested to mimic the in vivo condition of multilayers of TM cells in the TM. The effects of Dex and Rip were evaluated over 6 days by treating them with serial concentrations of Dex in the presence or absence of Rip. As shown in Figure 5, the changes in TEER values of TM cells treated with serial concentrations of Dex increased in a dose-dependent manner (control vs. 10-nM Dex, *P* = 0.00053542; control vs. 100-nM Dex, *P* = 1.3467E-05; control vs. 10-µM Dex, *P* = 1.4478E-06) compared to the control groups. However, the TEER values significantly decreased for co-treatment with Dex+Rip (100-nM Dex vs. 100-nM co-delivery, *P* = 8.5322E-05; 10-µM Dex vs. 10-µM co-delivery, *P* = 1.1351E-06; control vs. 10-nM co-delivery, *P* = 3.6418E-05; control vs. 100-nM co-delivery, *P* = 0.00013671; control vs. 10-µM co-delivery, *P* = 0.00015127). Interestingly, the TEER values of 10-µM Rip increased statistically compared to the control groups (control vs. 10-µM Rip, *P* = 2.726E-5). However, the TEER values are still statistically less than the 100-nM Dex and 10-µm Dex treatment groups (100-nM Dex vs. 10-µM Rip, *P* = 0.0013; 10-µM Dex vs. 10-µM Rip, *P* = 2.66131E-06). Furthermore, co-delivery of Dex and Rip showed values comparable to those of the Rip group only near 170 (10-nM co-delivery vs. 10-µM Rip, *P* = 0.0352; 100-nM co-delivery vs. 10-µM Rip, *P* = 0.0048; 10-µM co-delivery vs. 10-µM Rip, *P* = 0.000127), suggesting that co-delivery of Rip with Dex completely reduced cell resistance to the Rip-only level.

**Figure 6.**
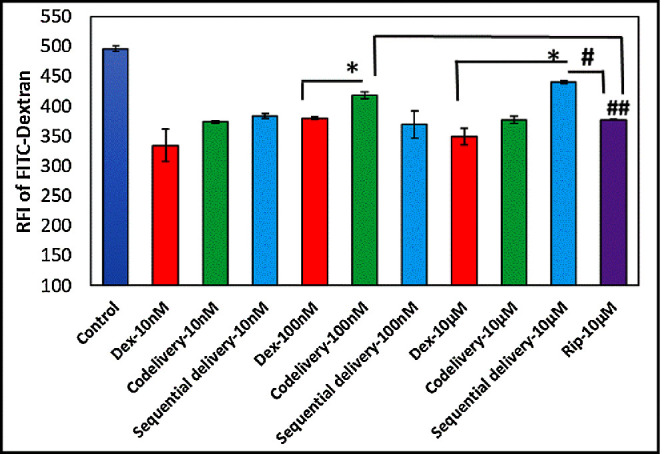
Dose-dependent effect of Dex and Rip on TM cell permeability. All data are presented as mean ± SD. ^*(#)^*P* < 0.05, ^**(##)^*P* < 0.01, ^***(###)^*P* < 0.001 compared to the control group or among the treatment groups (*n* = 4).

Similarly, to assess the TM cell monolayer barrier function, FITC–dextran permeability was measured. As shown in Figure 6, treatment of the TM cell monolayers with a serial concentration of Dex for 6 days resulted in a significant decrease in FITC–dextran permeability compared to the control groups (control vs. 10-nM Dex, *P* = 0.027534; control vs. 100-nM Dex, *P* = 0.002083; control vs. 10-µM Dex, *P* = 0.009337). On the other hand, FITC–dextran permeability increased significantly after co-delivery (100-nM Dex vs. 100-nM co-delivery, *P* = 0.020083) and sequential treatments with Dex and Rip (10-µm Dex vs. 10-µm sequential delivery, *P* = 0.0212). However, the Rip increments were not comparable to those of the control, suggesting that the permeability of FITC–dextran (MW 4000) was not completely restored by Rip. Nevertheless, 10-µM Rip only reduced the FITC–dextran permeability significantly compared to the control, indicating that Rip negatively affects the permeability of big molecules. Interestingly, 100-nM co-delivery (100-nM co-delivery vs. 10-µM Rip, *P* = 0.016) and 10-µM sequential delivery (10-µM sequential delivery vs. 10-µM Rip-10, *P* = 0.002) showed a statistically significant increase compared to 10-µM Rip.

## Discussion

We observed bright F-actin staining signals in Dex-treated TM cells, in a dose-dependent manner compared to control groups. However, the F-actin staining signal was decreased in TM cells treated with Dex+Rip. Moreover, Dex-treated TM cells showed well-aligned/straight-shaped polymerized F-actin/stress fibers, whereas Dex+Rip-treated TM cells showed loose and crooked-shaped F-actin polymerization. These results suggest that Dex enhances F-actin polymerization, whereas Rip inhibits F-actin polymerization or facilitates F-actin depolymerization.^29^ These findings demonstrate that Rip reverses the effects of Dex on the cytoskeleton rearrangement of TM cells when both are present. Interestingly, with Rip treatment, the fiber shape became loose and crooked, compared to the control.

Numerous in vitro experiments have demonstrated that the TM is a contractile tissue that is partly controlled by F-actin.^30^ F-actin is a major component of the cytoskeleton that is organized to regulate cell contraction. There are various culture models that can be used to investigate the ability of cells to reorganize and contract collagen matrices in vitro. The collagen gel contraction assay is a valuable tool for identifying novel mediators of TM cell relaxation. This is because contraction of the TM cells reduces the permeability of the TM, resulting in a decrease in the size of the intercellular spaces and a subsequent reduction in AH outflow.^31^^,^^32^

In this study, we used a two-step collagen contraction model to investigate the effect of Dex, Rip, and Dex+Rip on TM cell–mediated collagen contraction. The two-step model involves an initial period of attached matrix contraction that leads to mechanical loading, followed by the release of the matrices, resulting in mechanical unloading and further contraction as mechanical stress dissipates. Our cell contractility assay results showed that Dex induced marked gel contraction compared to the control group. Additionally, the collagen gel contraction activity of TM cells was significantly inhibited by co-delivery (Dex+Rip) or sequential addition of Rip on Dex-pretreated TM cell–collagen gel matrix. Overall, our findings suggest that Rip can significantly inhibit collagen contraction by TM cells or even reverse Dex-induced TM cell collagen gel contraction, for both co-delivery and sequential treatment. Furthermore, we observed that dosing polymerized collagen gel with Dex or Rip had no effect on gel contraction in the absence of TM cells, indicating that TM cells are contractile tissues themselves and that Dex only enhances their contractility ability. Therefore, the results also suggest that delivering Rip while treating with Dex may be crucial in reducing and/or preventing the Dex-induced elevated IOP, a risk factor for glaucoma formation. Overall, this study suggests that Rip may not completely prevent stress fiber formation but does to some extent and reduces contraction by tight and straight stress fibers.

TEER measurement and permeability assays are commonly used methods for evaluating the integrity of tight junction dynamics in the cell culture models of endothelial and epithelial monolayers.^33^ In this study, we observed that TEER values of TM cells increased in a dose-dependent manner when treated with serial concentrations of Dex. However, in TM cells dosed with Dex+Rip, the TEER values were significantly decreased. These results suggest that Rip has the potential to counteract the increase in AH outflow resistance induced by Dex, which could play a significant role in reducing IOP. In addition, our study demonstrated that the use of Dex resulted in a significant decrease in FITC–dextran permeability through the TM cell monolayer compared to the control group, where only the medium was used. The FITC–dextran permeability test with Rip showed inconsistent results among the various concentrations of Dex, unlike the TEER measurements, indicating that permeability may depend on the size of molecule that penetrates the cells. In other words, the AH outflow resistance can be restored by Rip for small molecules such as water but not for larger molecules such as FITC–dextran 4000. This is consistent with previous research indicating that Rip affects TM cellular shape, adhesion, migration, and F-actin cytoskeletal organization, which subsequently leads to the relaxation of multiple points of resistance within the TM cells.^34^^–^^38^ In summary, our findings suggest that Rip and Dex have opposing effects on the morphology of TM cells and consequently on the permeability of the tissue to AH outflow. Therefore, delivering Rip while treating with Dex may be a good strategy to prevent Dex-induced elevated IOP, a major risk factor for glaucoma formation.

Finally, although ROCK inhibitors showed a promising safety profile, several studies have reported that they have both local and systemic adverse effects such as conjunctival hyperemia and subconjunctival hemorrhage due to their vasodilatory effect.^39^^–^^41^ The latter may increase the clearance of concomitantly administered topical drugs, thereby reducing their intended ocular effects.^42^ Other local effects include blepharitis, ocular irritation, increased lacrimation, and blurred vision. On the systemic level, they may cause blood pressure reduction and an associated increase in heart rate.^43^

There are some limitations associated with the use of cell cultures to study Dex-induced elevated IOP. Due to the complexity of the TM tissue and its interactions within the multiple ocular structures to regulate IOP, isolating single TM cells and culturing them on two-dimensional culture dishes may not accurately represent these interactions. In addition, because glaucoma is a chronic disease that develops over years, long-term studies are needed to identify the gradual progression of glaucoma, as it is difficult to determine that with the use of short-term in vitro cell models (for example, we dosed only for 6 days in the current experiments).

## Conclusions

Herein, the study aimed to evaluate the impact of Dex, Rip, and Dex+Rip on trabecular meshwork cells. Our findings demonstrate that Rip has the ability to reverse the morphological and biochemical alterations induced by Dex, such as F-actin organization, collagen gel contraction, and transepithelial electrical resistance, in cultured trabecular meshwork cells. These observations suggest that combining Dex and Rip may be critical in promoting aqueous humor outflow, which can help prevent the formation of intraocular pressure and, ultimately, the development of glaucoma.

## Supplementary Material

Supplement 1
