# Supplementary material for: The Effects of ROCK Inhibitor on Prevention of Dexamethasone-Induced Glaucoma Phenotype in Human Trabecular Meshwork Cells
Source: Transl Vis Sci Technol. 2023 Dec 5;12(12):4. doi: 10.1167/tvst.12.12.4 (PMC10702786; doi:10.1167/tvst.12.12.4)
Supplement: Supplement 1 [file tvst-12-12-4_s001.pdf]

The Effects of ROCK Inhibitor On Prevention Of Dexamethasone-Induced Glaucoma Phenotype In Human Trabecular Meshwork Cells

Tilahun Ayane Debele, Zachary Mount, Yong Yuan, Winston W-Y. Kao and Yoonjee C. Park

Department of Chemical & Environmental Engineering, College of Engineering and Applied Science (CEAS), University of Cincinnati, Cincinnati, OH 45221, USA

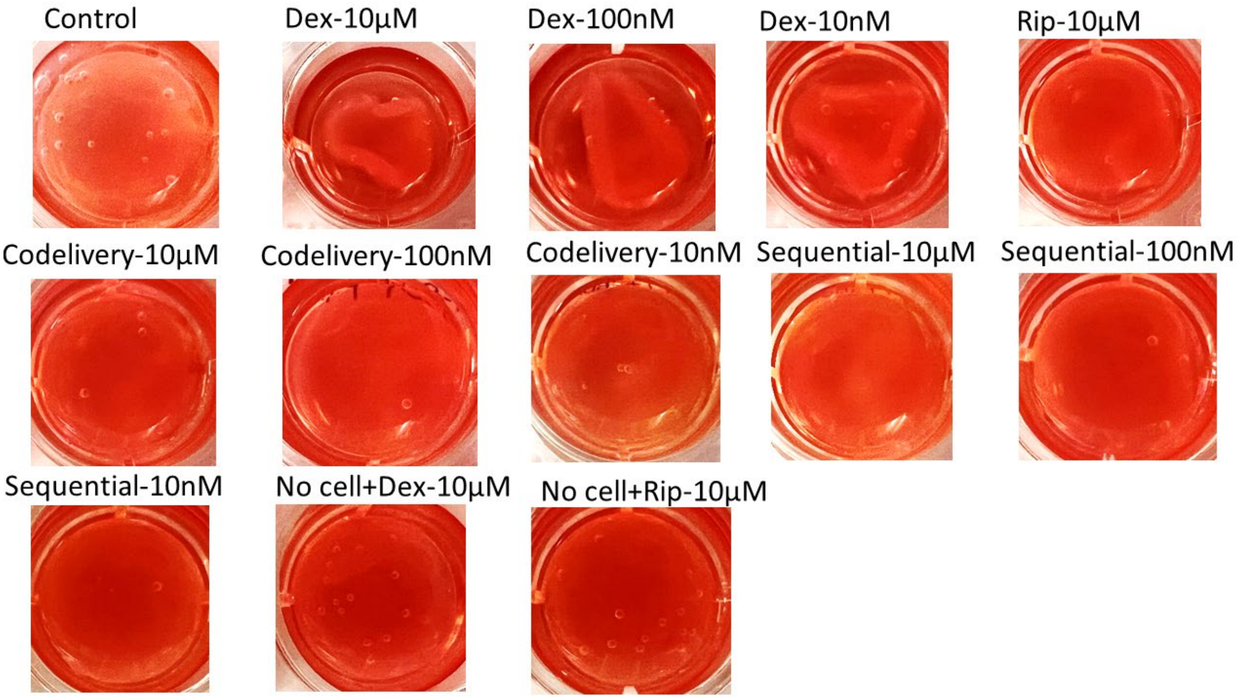

Figure S1. Representative image of TM cells collagen gel matrix treated with Medium (control), Rip, Dex, co-delivery (Dex+Rip), Sequential treatment (the concentration in the codelivery and sequential treatment is 10 μM), No TM cell + Dex 10 μM and No TM cell +Rip μM.

The summary of calculated P-value using on-tailed student T-test**.** P-value <0.05 is considered statistically significant. The significance level was represented using asterisks as follows: * for p-value < 0.05, ** for p-value < 0.01, and *** for p-value < 0.001. NS: Not Significant.

Table S1. Summary of calculated P-value for MTT cytotoxicity assay.

**Comparison**	**P-value**	**Statistical significance**
Control vs Dex-10nM	0.132018	NS
Control vs Dex-100nM	0.476581	NS
Control vs Dex-1μM	0.274587	NS

Control vs Dex-10µM	0.281774	NS
Control vs Dex-20µM	**0.035731**	*
Dex-10nM vs Dex-100nM	0.267108	NS
Dex-10nM vs Dex-1µM	0.067268	NS
Dex-10nM vs Dex-10µM	0.281774	NS
Dex-10nM vs Dex-20µM	0.035731	*
Dex-100nM vs Dex-1µM	0.338177	NS
Dex-100nM vs Dex-10µM	0.332144	NS
Dex-100nM vs Dex-20µM	0.145185	NS
Dex-1µM vs Dex-10µM	0.47646	NS
Dex-1µM vs Dex-20µM	0.137635	NS
Dex-10µM vs Dex-20µM	0.195231	NS
Control vs Rip-0.5µM	0.483543	NS
Control vs Rip-1µM	0.036855	*
Control vs Rip-5µM	0.058329	NS
Control vs Rip-10µM	0.310673	NS
Control vs Rip-20µM	0.499308	NS
Rip-0.5µM vs Rip-1µM	0.093958	NS
Rip-0.5µM vs Rip-5µM	0.106905	NS
Rip-0.5µM vs Rip-10µM	0.318341	NS
Rip-0.5µM vs Rip-20µM	0.480476	NS
Rip-1µM vs Rip-5µM	0.469052	NS
Rip-1µM vs Rip-10µM	0.121909	NS
Rip-1µM vs Rip-20µM	0.053086	NS
Rip-5µM vs Rip-10µM	0.146707	NS
Rip-5µM vs Rip-20µM	0.066616	NS
Rip-10µM vs Rip-20µM	0.301672	NS
Control vs Codelivery-10nM	0.404712	NS
Control vs Codelivery -100nM	0.046934	*
Control vs Codelivery -1µM	0.37468	NS
Control vs Codelivery -10µM	0.015242	*
Control vs Codelivery -20µM	0.032135	*
Codelivery -10nM vs Codelivery -100nM	0.156017	NS
Codelivery 10nM vs Codelivery -1µM	0.465501	NS
Codelivery -10nM vs Codelivery -10µM	0.096152	NS
Codelivery 10nM vs Codelivery -20µM	0.147664	NS
Codelivery -100nM vs Codelivery -1µM	0.203634	NS
Codelivery -100nM vs Codelivery -10µM	0.353725	NS
Codelivery -100nM vs Codelivery -20µM	0.485461	NS
Codelivery -1µM vs Codelivery -10µM	0.138305	NS
Codelivery -1µM vs Codelivery -20µM	0.198575	NS
Codelivery -10µM vs Codelivery -20µM	0.31775	NS

Table S2. Summary of calculated P-value for mean gray value of F-actin cytoskeleton staining.

**Comparison**	**P-value**	**Statistical significance**
Control VS Dex-10nM	0.073993767	NS
**Control VS Dex-10nM+Rip10μM**	**0.005555047**	**
Control VS Dex-100nM	0.272756137	NS
**Control VS 100nM+Rip10μM**	**0.000505806**	***
**Control VS Dex-10μM**	**0.008561928**	**
Control VS Dex-10μM+Rip10μM	0.343617882	NS
Control vs Rip-10μM	0.418220536	NS
**Dex-10nM vs Dex-10nM+Rip10μM**	**0.002583033**	**
Dex-10nM VS Dex-100nM	0.192360868	NS
Dex-10nM VS Dex-10μM	0.136776335	NS
**Dex-100nM vs Dex-100nM+Rip10μM**	**0.001292567**	**
**Dex-100nM VS Dex-10μM**	**0.030814725**	*
**Dex-10μM VS Dex-10μM+Rip10μM**	**0.008310712**	**
Dex-10nM VS Rip-10μM	0.068363485	NS
Dex-10μM VS Rip-10μM	0.009049204	**
Dex-100nM VS Rip-10μM	0.235569644	NS

Table S3. Summary of calculated P-value for Collagen gel area.

**Comparison**	**P-value**	**Statistical significance**
**Control VS Dex-10nM**	**0.168055**	**NS**
Control VS Codelivery-10nM	0.466313	NS
Control VS Sequential delivery-10nM	0.357137	NS
**Control VS Dex-100nM**	**0.103836**	**NS**
Control VS Codelivery-100nM	0.210352	NS
Control VS Sequential delivery-100nM	0.495536	NS
**Control VS Dex-10μM**	**0.136496**	**NS**
Control VS Codelivery-10μM	0.331643	NS
Control VS Sequential delivery-10μM	0.230105	NS
**Control VS Rip-10μM**	**0.3458**	**NS**
**Dex-10nM vs Codelivery-10nM**	**0.012295**	*
**Dex-10nM VS Sequential delivery-10nM**	**0.00105**	**
**Dex-10nM VS Dex-100nM**	**0.055396**	**NS**
**Dex-10nM VS Dex-10μM**	**0.133696**	**NS**
**Dex-100nM vs Codelivery-100nM**	**0.003138**	**
**Dex-100nM VS Sequential delivery-100nM**	**0.0493**	*

**Dex-100nM VS Dex-10µM**	**0.157425**	**NS**
**Dex-10µM vs Codelivery-10µM**	**0.028513**	*****
**Dex-10µM vs Sequential delivery-10µM**	**0.009163**	******
Dex-10µM vs Rip-10µM	0.181237	NS
Dex-100nM vs Rip-10µM	0.125574	NS
Dex-10nM vs Rip-10µM	0.239953	NS

Table S4. Summary of calculated P-value for TEER measurements.

Comparison	P-value	Statistical significance
Control vs Dex-10nM	0.00053542	***
Control vs Dex-100nM	1.3467E-05	***
Control vs Dex-10µM	1.4478E-06	***
Control vs Rip-10µM	2.726E-05	***
Control vs Codelivery-10nM	3.6418E-05	***
Control vs Codelivery-100nM	0.00013671	***
Control vs Codelivery-10µM	0.00015127	***
Dex-10nM vs Dex-10nM+Rip-10µM	0.37559092	NS
Dex-10nM vs Dex-100nM	0.00082064	***
Dex-10nM vs Dex-10µM	7.9216E-05	***
Dex-100nM vs Codelivery-100nM	8.5322E-05	***
Dex-100nM vs Dex-10µM	0.00109606	***
Dex-10µM VS Codelivery-10µM	1.1351E-06	***
Dex-10nM vs Rip-10µM	0.37559092	NS
Dex-100nM vs Rip-10µM	0.00013062	***
Dex-10µM vs Rip-10µM	2.6613E-06	***
Codelivery-10nM VS Rip-10µM	0.035242	*
Codelivery-100nM VS Rip-10µM	0.00483948	**
Codelivery-10µM VS Rip-10µM	0.00012761	***

Table S5. Summary of calculated P-value for permeability measurements.

Comparison	P-value	Statistical significance
Control vs Dex-10nM	0.027534	*
Control vs Codelivery-10nM	0.001999	**
Control vs Sequential delivery-10nM	0.003565	**
Control vs Dex-100nM	0.002083	**
Control vs Codelivery-100nM	0.008111	**
Control vs Sequential delivery-100nM	0.030163	*
Control vs Dex-10µM	0.009337	**
Control vs Codelivery-10µM	0.004766	**
Control vs Sequential delivery-10µM	0.00995	*
Control vs Rip-10µM	0.001796	**

Dex-10nM vs Rip-10µM	0.193757	NS
Codelivery-10nM vs Rip-10µM	0.207314	NS
Sequential delivery-10nM vs Rip-10µM	0.227265	NS
Dex-100nM vs Rip-10µM	0.231729	NS
Codelivery-100nM vs Rip-10µM	0.016024	*
Sequential delivery-100nM vs Rip-10µM	0.412447	NS
Dex-10µM vRip-10µM	0.138578	NS
Codelivery-10µM vs Rip-10µM	0.499721	NS
Sequential delivery-10µM vs Rip-10µM	0.002044	**
Dex-10nM vs Codelivery-10nM	0.212484	NS
Dex-10nM vs Sequential delivery-10nM	0.17066	NS
Dex-100nM vs Codelivery-100nM	0.020083	*
Dex-100nM vs Sequential delivery-100nM	0.383172	NS
Dex-10µM vs Codelivery-10µM	0.15694	NS
Dex-10µM vs Sequential delivery-10µM	0.0212	*
Dex-10µM vs Dex-10nM	0.387127	NS
Dex-10µM vs Dex-100nM	0.123769	NS
Dex-100nM vs Dex-10nM	0.181793	NS
